# Mindfulness-Based Cognitive Therapy for Life (MBCT-L) Versus Stress Reduction Psychoeducation (SRP) for the Improvement of Mental Well-Being in Health Care and Other Public Sector Staff: Protocol for the Well at Work Randomized Controlled Trial

**DOI:** 10.2196/67695

**Published:** 2025-05-26

**Authors:** Elena Nixon, Shireen Patel, Priya Patel, James Roe, Neil Nixon, Tim Sweeney, Paul Bernard, Clara Strauss, Michael P Craven, Sam Malins, Rob Goodwin, Laurence Astill Wright, Boliang Guo, Richard Morriss

**Affiliations:** 1 Mental Health and Clinical Neurosciences Institute of Mental Health Nottingham United Kingdom; 2 Nottinghamshire Healthcare NHS Foundation Trust Nottingham United Kingdom; 3 Tees, Esk and Wear Valleys NHS Foundation Trust Darlington United Kingdom; 4 Sussex Partnership NHS Foundation Trust Hove United Kingdom; 5 School of Psychology University of Sussex Brighton United Kingdom; 6 Faculty of Engineering University of Nottingham Nottingham United Kingdom; 7 Injury, Recovery and Inflammation Services School of Medicine University of Nottingham Nottingham United Kingdom; 8 Centre for Academic Mental Health Population Health Sciences University of Bristol Bristol United Kingdom

**Keywords:** health care, National Health Service, NHS, staff, digital intervention, staff well-being, Mindfulness-Based Cognitive Therapy, therapy, Stress Reduction Psychoeducation, randomized controlled trial

## Abstract

**Background:**

Mindfulness-based and stress reduction interventions have been recommended by the National Institute for Health and Care Excellence guidelines in England and Wales as effective preventive mental well-being interventions for health care and other public sector staff at risk of poor mental health.

**Objective:**

This trial aims to assess the effectiveness of the increasingly implemented Mindfulness-Based Cognitive Therapy for Life (MBCT-L) intervention versus a routinely available Stress Reduction Psychoeducation (SRP) intervention in reducing perceived stress and improving other mental health and work-related outcomes in national health care and other public sector service employees.

**Methods:**

The trial is a multisite, single-blind, parallel-group, 2-arm superiority randomized controlled trial. Recruitment, interventions, and assessments will be conducted remotely via online platforms. We will recruit 260 health care and other public sector staff into 26 intervention groups across the United Kingdom, with the intervention delivered through human resource staff well-being provision channels affiliated with participating National Health Service trusts. Participants will be randomly allocated in a 1:1 ratio to either MBCT-L or SRP. Primary and secondary outcomes will be collected at 6, 12, and 20 weeks after randomization. The primary outcome will be the change in scores on the Perceived Stress Scale-14 from baseline to 20 weeks after randomization. Demographic, intervention-related, and health economic data will also be collected. Secondary outcomes will involve assessments of well-being, mental health state, and work-related engagement and performance. Adverse events will be recorded. Data analysis will involve multilevel modeling, and it will be conducted on an intention-to-treat basis. A substudy will involve online semistructured interviews after 20 weeks of randomization with a subsample of participants (n=30, 12%). Transcribed data will be subjected to thematic analysis to elicit qualitative outcomes on perceived well-being and work-related changes after intervention as well as drivers and barriers to intervention uptake and acceptability.

**Results:**

Recruitment of participants commenced on August 29, 2023. The target recruitment of 260 participants was reached on April 30, 2024. Follow-up outcome data collection was completed on September 30, 2024, and data analysis is underway. A total of 30 qualitative interviews have been conducted.

**Conclusions:**

Findings will inform future recommendations on intervention suitability and implementation for public care staff well-being.

**Trial Registration:**

International Standard Randomised Controlled Trial Number (ISRCTN) ISRCTN18049845; https://www.isrctn.com/ISRCTN18049845

**International Registered Report Identifier (IRRID):**

DERR1-10.2196/67695

## Introduction

### Background

Public sector employees present with higher levels of stress compared to those in other job sectors, with health care and public care staff, in particular, experiencing disproportionately high stress levels [1]. Stress levels in public sector employees have been further exacerbated by the COVID-19 pandemic, which has reportedly imposed additional stressors in the workplace, especially on health care staff [2-4]. In 2020-2021, stress, depression, or anxiety accounted for 50% of all work-related ill health, with the total number of cases of work-related stress, depression, or anxiety being 822,000, and a prevalence rate of 2480 per 100,000 employees in human health, social care, and education sectors in the United Kingdom [1]. Excessive stress and mental health problems, such as anxiety and depression, in public sector staff have also been broadly associated with further negative individual-level outcomes, such as burnout, compassion fatigue, and reduced quality of life and negative organizational outcomes, such as poor job satisfaction; presenteeism; and absenteeism and leaving work, including poor care provision [5-7]. Mindfulness-based training and stress management training are two approaches that have been recently recommended by the National Institute for Health and Care Excellence (NICE) [8] as effective psychotherapeutic interventions to be delivered in the United Kingdom in either online or face-to-face group modality to health care or other public care employees at risk of poor mental health.

While a broad range of organizational- and individual-level well-being interventions are currently being offered in National Health Service (NHS) trusts and other public sector workplaces, Stress Reduction Psychoeducation (SRP) is a standard usual care group program offered widely and in various formats across all geographic regions in England (United Kingdom) to health care staff as well as other public sector staff accessing psychotherapeutic interventions through their organizational well-being services affiliated with NHS trusts. Mindfulness-Based Cognitive Therapy for Life (MBCT-L) [9] is a newer, third-wave intervention, which is increasingly being implemented in the NHS and other public sector services. MBCT-L borrows its premises from the standard Mindfulness-Based Cognitive Therapy (MBCT) variant [10,11], which is a NICE-recommended treatment option for recurrent and mild to moderate acute depression [12], and is also included in clinical guidelines as a relapse prevention option in other countries [13-15]. Importantly, MBCT-L is an adaptation tailored to nonclinical populations and has been shown to be effective in improving well-being and mental health outcomes in various populations, including health care workers, as evidenced in previous work [16-20], including a recent randomized controlled trial (RCT) that showed moderate to large effects on health care staff well-being derived from MBCT-L compared to a waitlist control [21].

SRP and MBCT-L approaches are increasingly being implemented across the NHS trusts in the United Kingdom in online formats, particularly after the COVID-19 pandemic, in acknowledgment that digital modalities can overcome main barriers to intervention uptake and adherence [22]. However, MBCT-L is currently being offered as a workplace psychological program through the NHS trust’s well-being services in a limited number of trusts across the United Kingdom. Notably, MBCT-L is a longer-duration program, commonly lasting 8 or 9 weeks if a practice (retreat) day is included, as opposed to SRP, the duration of which can vary between 4 and 6 weeks. Furthermore, MBCT-L requires more skilled staff for its delivery, that is, specifically MBCT-trained staff, whereas SRP programs can be delivered by classically trained cognitive behavioral therapy practitioners, psychological well-being practitioners, or other health care staff trained in supporting people with common mental health problems in managing their conditions.

SRP and MBCT-L approaches differ in their conceptual and practical premises; while SRP relies on behavioral stress reduction techniques coupled with well-being and goal setting [23], MBCT-L is an enhanced approach that combines both stress reduction and cognitive therapy elements in one program, embedding mindfulness practices as well [9]. Thus, a presumed advantage of MBCT-L over SRP approaches is that the former teaches foundational coping skills that not only alleviate transient stress but also cultivate a long-term change in one’s attitude toward stress and promote a more positive outlook on stressful experiences and life more generally.

However, the 2 approaches have not yet been compared to one another in terms of their effectiveness as workplace interventions in improving stress and other well-being aspects and job-related outcomes. The findings of the review conducted by NICE in their recent guidance [8] indicated larger effects of face-to-face mindfulness-based approaches on stress and other mental health and job-related outcomes than stress reduction interventions in at-risk public sector populations. Moderate-quality evidence suggested that mindfulness-based approaches are effective in improving mental well-being; managing mental health symptoms; and decreasing absenteeism in public sector populations, including health care staff. Very-low-quality evidence suggested that such mindfulness interventions may improve job stress in such at-risk public sector populations. Furthermore, the reviewed mindfulness-based studies seemed to rely on mindfulness-based stress reduction approaches, largely without cognitive elements embedded, while some of the reviewed SRP studies included cognitive elements in their content and exercises. As for stress reduction approaches, moderate-quality evidence indicated that SRP was effective in improving job satisfaction; quality of life; and mental health literacy in targeted high-risk populations, including health care staff. Low-quality and very-low-quality evidence hinted that a stress management approach might be effective in improving stress and mental health symptoms in these populations.

### Study Rationale

The NICE review did not include any online mindfulness-based studies, while the included stress management interventions were delivered either face-to-face or online. All 5 mindfulness-based studies and all 5 stress management studies included in the NICE review had a waitlist or usual care as a comparator or no well-being comparator intervention. Only 1 of the stress management studies was based in the United Kingdom, while there were no other mindfulness-based studies that were conducted in the United Kingdom. Finally, while the NICE review consolidated some qualitative data assessing barriers and facilitators to intervention uptake, it only included 1 qualitative study, which involved another type of intervention, that is, digital cognitive behavioral therapy [8]. The findings of the study indicated that there are a number of positive aspects of the digital modality of such a well-being intervention, including convenience and discreteness or anonymity, while specific barriers (eg, time pressures) and drivers (eg, managerial support) were reported to impact engagement with the given intervention.

Therefore, in light of the lack of online mindfulness-based studies in the NICE review and a lack of studies directly comparing mindfulness-based and SRP approaches, the proposed superiority trial will assess the effectiveness and cost-effectiveness of the 2 approaches. Furthermore, in light of the lack of qualitative studies exploring the acceptability or effectiveness of either approach, this study aims to gather in-depth qualitative data from a subsample of participants to explore the perceived impact of the intervention, including postintervention changes in well-being and work performance, barriers and facilitators to the uptake and acceptability of such interventions, and their engagement throughout the program. The overarching research question is as follows: Does MBCT-L demonstrate superior efficacy to SRP in reducing perceived stress and improving mental health and work-related outcomes among public sector employees? The findings of this study are expected to fill in the aforementioned gaps in the existing research and are intended to inform the NHS and other public sector organizations on how to best invest in their future resource provision to retain and support their staff at risk of poor well-being.

## Methods

### Aim, Design, and Setting of the Study

#### Overview

The proposed Well at Work study is a single-blind, multisite, parallel-group, 2-arm superiority RCT that aims to assess the clinical effectiveness and cost-effectiveness of MBCT-L versus SRP in health care and other public sector staff who are seeking to access well-being support through human resource channels affiliated with the participating NHS trusts. The primary objective of this trial will be to determine whether there is a difference in change in the primary outcome on the Perceived Stress Scale-14 (PSS-14 [24]) between the 2 intervention groups from baseline to 20 weeks after randomization. The secondary objectives will be to assess the differences in change in any of the secondary mental well-being and work-related outcome measures between the 2 arms from baseline to each follow-up time point, that is, at 6, 12, and 20 weeks after randomization. Recruitment, interventions, and assessments will be conducted remotely via online platforms using videoconferencing and electronic technologies.

#### Qualitative Study

A substudy will involve online semistructured interviews to be conducted 20 weeks after randomization, with a subsample of participants from each arm gathering qualitative data for in-depth insights into participants’ perceived changes after intervention and their experiences and views in relation to intervention uptake and acceptability.

The Well at Work study is hosted by the University of Nottingham in the United Kingdom, which is the sponsor of the study, and will be mainly based across 4 public care (NHS) sites over 3 geographic regions in the United Kingdom, that is, Nottingham University Hospitals NHS Trust and Nottinghamshire Healthcare NHS Foundation Trust (the East of England); Sussex Partnership NHS Foundation Trust (South of England); and Tees, Esk and Wear Valleys NHS Foundation Trust (North of England). The online MBCT-L intervention will be offered across all 4 NHS trust sites, while 9 additional NHS trust sites will also be participating in the trial, namely Lincolnshire Partnership NHS Foundation Trust; Lincolnshire Community Health Services NHS Trust; Derbyshire Community Health Services NHS Foundation Trust; York and Scarborough Teaching Hospitals NHS Foundation Trust; South Tyneside and Sunderland NHS Foundation Trust; Leicestershire Partnership NHS Trust; Wrightington, Wigan and Leigh Teaching Hospitals NHS Foundation Trust; South West London and St George’s Mental Health NHS Trust; and Northern Care Alliance NHS Foundation Trust. These additional sites will be channeling interested staff to 1 of the 4 main NHS trust sites delivering the interventions. Given the online intervention delivery format, the trial aims, with this approach, to achieve wide geographic coverage and outreach across health care and other public sector staff in the United Kingdom seeking well-being support through their organizations, which is offered via the participating NHS trust sites.

#### Patient and Public Involvement and Engagement

A patient and public involvement and engagement (PPIE) group consisting of members of staff working in the public sector has been formed and consulted on the design of the study. The PPIE group is intended to provide input into all key aspects of the trial throughout its duration, from conceptualization to implementation, evaluation, and dissemination.

Reporting of the protocol is according to SPIRIT (Standard Protocol Items: Recommendations for Interventional Trials) guidelines (Multimedia Appendix 1).

### Sample Selection

Potential participants will be directed to the study through existing integrated care services; well-being hubs; and other human resource channels, which are accessed by staff working in health care and other public sectors, for example, social care and teaching, through the NHS trust sites participating in the trial. The communication of study information will be facilitated through a study flyer that will be circulated widely across the participating sites via email and other communication channels, including staff communication hubs; staff well-being websites; staff networks, newsletters, and social media (eg, Facebook [Meta Platforms, Inc] and X [X Corp]); and staff induction or other events.

Staff expressing an interest in the study will be directed to an online link facilitated via REDCap (Research Electronic Data Capture; Vanderbilt University) [25,26] tools hosted at the University of Nottingham, which will include a participant information sheet (PIS) with information about the study, followed by an eligibility screening form. The contact details of the study researchers will be provided in the PIS should any interested participants wish to ask any questions. Upon completion of the eligibility form, eligible participants will be emailed a link to a participant consent form on the REDCap platform. In the consent form, participants will also be asked to indicate whether they would be willing to receive information at a later stage about the qualitative substudy. Following the provision of informed consent on REDCap, participants will be directed on the platform to the survey containing the baseline assessment questionnaires. The inclusion and exclusion criteria are listed in Textbox 1.

**Inclusion and exclusion criteria for the trial.**

**Inclusion criteria**

- Aged ≥18 years
- Part-time, full-time, honorary, or voluntary employment, seeking access to well-being support through one of the participating sites
- Currently working (ie, not on a sick leave)
- Having a competent command of the verbal and written English language
- Having access to a stable internet connection on a PC, laptop, or tablet

**Exclusion criteria**

- Current diagnosis of a mental health condition from a general practitioner or mental health care professional
- Experience of significant life events currently causing significant distress
- Concurrently attending or planning to attend a psychological or well-being intervention within the subsequent 3 months

### Screening

Exclusion criteria will be flagged up in the screening form, and interested participants deemed as ineligible will be contacted by the trial practitioner or facilitator team to verify their eligibility; if exclusion criteria are confirmed, interested participants will be signposted to alternative available support as appropriate, for example, to an MBCT for depression program run routinely at each involved site if participants experience high levels of depression. As this is the mechanism already in routine place for staff recruitment for these interventions through NHS trust well-being services, we will keep our exclusion criteria to the minimum.

### Sample Size

With reference to the trial’s primary outcome, 5 points on the PSS-14 will be considered as the minimum clinically important difference. Assuming a 2-tailed .05 significance level with 90% power (using analysis of covariance) and SD 7.7, a baseline and follow-up correlation of 0.2, and the correlation among follow-up measures of 0.7, a total of 76 participants will be needed to estimate the treatment effect from baseline at 20 weeks after randomization. Assuming the average group size is 8, the intraclass correlation coefficient is 15%, and the attrition rate is 25% (considering potential loss to follow-up), the final sample size was inflated to 208 participants to be randomly recruited in 26 groups (1:1 ratio) across all the recruitment sites. However, upon monitoring follow-up rates after approximately one-third of the participants were randomized, it was highlighted by a blinded independent statistician that the follow-up rate was approximately 62% and the group size was approximately 10. Therefore, the trial management group decided to update the sample size with 85% power to mitigate the loss to follow-up. A substantial amendment was submitted, and ethics approval was received for the sample size to be increased to 260 participants, divided across 26 groups.

### Ethical Considerations

The trial has been granted ethics approval by the research ethics committee (East Midlands-Nottingham 1), the Health Research Authority, and the Health and Care Research Wales Committee (reference number 23/EM/0109) and has received favorable approvals from the research and development governance bodies of the participating NHS trust sites. The study has also been reviewed by the National Institute of Health and Care Research Applied Research Collaboration East Midlands (ARC-EM) Scientific Committee as part of the funding application.

The trial will be overseen by an independent ARC-EM Scientific Committee. The members of the committee will be drawn from outside the institutions where the research team currently works to ensure the committee’s independence from the research team. It will serve the function of a trial steering committee and a data monitoring committee. The trial will be reviewed every 6 months through reports to the ARC-EM Scientific Committee and presentations of progress to the committee by the study team. The data monitoring committee will review safety data and periodically review the conduct of the study. The steering committee will take responsibility for the scientific validity of the study protocol, assessment of study quality and conduct, and the scientific quality of the final study report. The sponsor and the investigators participating in the trial bear the final responsibility for the conduct of the trial. The trial will be conducted in accordance with the ethical principles that have their origin in the Declaration of Helsinki, the principles of Good Clinical Practice, and the UK Department of Health Policy Framework for Health and Social Care.

All participants will sign the participant consent form. Participants will be informed in the PIS that entry into the trial is entirely voluntary and that their health care, work, and legal rights will not be affected by their decision. Furthermore, it will be explained that they can withdraw at any time during the trial (including at follow-up) but that in the event of their withdrawal, data collected up to that point cannot be erased and may still be used in the final analysis. It will also be clearly stated in the PIS that potential participants who decide not to take part in the trial can proceed with enrolling in the respective or other well-being interventions delivered routinely at a given site (but are not part of the trial). Moreover, for intention-to-treat (ITT) purposes, as stated in the PIS, participants will have the right to decide not to withdraw from the trial entirely but withdraw from the program (intervention) while they may still contribute to the trial by completing the web-based survey at the 3 follow-up time points, or they can carry on with their participation in the program but withdraw their partaking in the completion of the web-based survey at the follow-up time points. Insurance and indemnity for trial participants and trial staff are covered within the NHS Indemnity Arrangements for clinical negligence claims in the NHS, issued under the cover of Health Systems Global 96/48. There are no special compensation arrangements, but trial participants may have recourse through the NHS complaint procedures. The University of Nottingham, as the research sponsor, indemnifies its staff with both public liability insurance and clinical trials insurance for claims made by research participants.

### Randomization and Blinding

Once consent is obtained on the REDCap platform, they will be randomly allocated in a 1:1 ratio to either the MBCT-L or SRP intervention. The online format and delivery of the interventions allow the allocation of participants to take place across the participating sites; this is expected to facilitate timely and sufficient recruitment within and across the intervention groups running at any site until the 2 intervention groups are full in each recruitment wave throughout the trial. Randomization will be conducted via a web-based randomization system set up by a clinical decision support system supported by the University of Nottingham. The randomization system will ensure that all the researchers involved in the outcome assessments (SP, PP, and JR) remain blinded to intervention allocation. Participant allocation to either intervention arm will be solely facilitated by a project administrator who will manage the randomization system independently of the research team following specified standard operating procedures. The study statistician will also be blinded to treatment allocation. In the event of inadvertent unblinding, the details of the unblinding incident will be recorded in the study log, relevant sponsor and monitoring committees will be informed (eg, the university sponsor, the research ethics committee, and the ARC-EM Scientific Committee), and the extent and impact of the unblinding will be assessed. All participants will complete the same outcome measures in the survey, so that researchers or outcome assessors will not be able to determine the participant’s group based on the response. In view of the nature of the interventions, participants cannot be blinded; they will be informed of which treatment arm they have been randomized to and will be notified through a letter or via email sent to them by the project administrator, which will also include information on the intervention delivery (eg, dates and times, requirements for attendance, and joining instructions).

### Interventions

#### Overview

**Figure 1:**
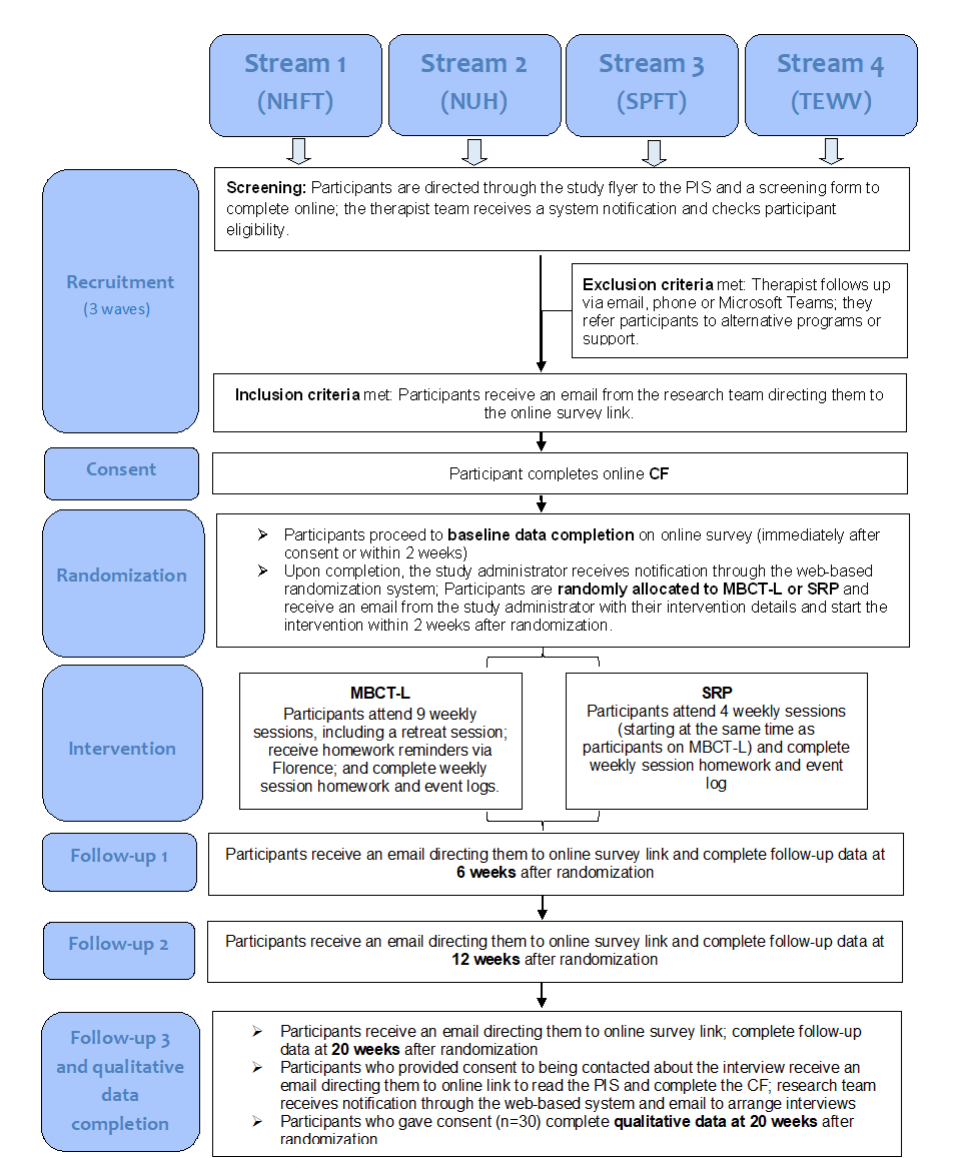
CONSORT (Consolidated Standards of Reporting Trials) flowchart—revised. CF: consent form; MBCT-L: Mindfulness-Based Cognitive Therapy for Life; NHFT: Nottinghamshire Healthcare National Health Service Foundation Trust; NUH: Nottingham University Hospitals; PIS: participant information sheet; SPFT: Sussex Partnership National Health Service Foundation Trust; SRP: Stress Reduction Psychoeducation; TEWV: Tees, Esk and Wear Valleys National Health Service Foundation Trust.

The 2 interventions that participants will be randomized, MBCT-L or SRP, will be delivered online via a videoconferencing platform, that is, Microsoft Teams, taking place in a group format once a week at different times and days, allowing participants to indicate (at the time of consent) which group times they would opt for if they were to be randomized to either intervention. The sessions on either intervention will not be recorded. There will be 3 recruitment waves in total, with the interventions starting at various time points during a given recruitment wave and both intervention arms starting in the same week in each recruitment wave to allow synchronized data collection. The CONSORT (Consolidated Standards of Reporting Trials) flowchart (Figure 1) depicts an outline of recruitment, consent, randomization, and assessment processes.

#### The MBCT-L Intervention

MBCT-L integrates conventional cognitive therapy techniques with stress reduction techniques and mindfulness practice, as in the original MBCT version, but has been adapted to apply to the general population as per established protocol [9]. The program includes 8 online weekly sessions lasting approximately 2 hours and an online practice day lasting approximately 6 hours. MBCT-L will run for 9 consecutive weeks or 10 weeks if a 1-week break is included (eg, due to school break or holidays) in a group-based format with an average of 8 participants recruited per group. The content includes themed teaching every week and guided meditation practices (eg, breathing practice and body scan), as well as everyday homework practices lasting 30 to 45 minutes. Participants will receive frequent automated text reminders about their upcoming sessions and homework or home practices via an automated text messaging service provided by a Generated Health system, Florence [27]. The program will be delivered by approximately 8 trained MBCT practitioners or facilitators across the main NHS trust sites, who are part of the teaching team involved in the MBCT-L interventions already being offered to public sector staff through the NHS trust services and have been trained under, and follow, the same MBCT training protocol [9].

The MBCT-L program broadly follows an overarching structure of developing mindfulness skills intended to enhance attentional control and awareness, cultivating a deeper understanding of how distress is created and maintained and how mindfulness training can address factors that contribute to its maintenance, learning skillful ways of relating to experience developed through awareness and practice, and learning to recognize unhelpful reactive patterns in everyday life and cultivate the capacity to respond to these with mindfulness and compassion. In the second part of the program, participants practice the application of this learning to everyday life, including work, to respond more effectively to stress and enjoy the positive aspects of life.

#### The SRP Intervention

The SRP intervention will be delivered in 4 online weekly sessions lasting approximately 2 hours and running for 4 consecutive weeks or 5 weeks if there is a 1-week break (eg, school break or other holidays). The program will run as it is currently being offered as standard care across Nottinghamshire and all the other regions in England, through NHS Talking Therapies (formerly known as Improving Access to Psychological Therapies) or NHS trust or other health care services. Its content is focused on psychoeducation around well-being and stress management, combined with relaxation strategies [23]. Specifically, the program consists of sessions focused on areas of stress, well-being, and goal setting; sleep hygiene; anxiety and depression; behavioral activation; problem-solving; thinking errors; and a final recap, review of goals and thoughts for future well-being. Participants will be encouraged to work between sessions by engaging in a stress reduction technique of their preference daily for approximately 30 minutes and through a diary activity (eg, behavioral activation diary). They will receive email reminders to complete these and attend the upcoming sessions.

Recognizing that SRP programs can vary in their content and format or structure in how they are being offered across NHS trusts and other organizations in the United Kingdom, the standard 4-session structure will be adopted as the optimized form of its delivery. The SRP will also explicitly avoid embedding any mindfulness-based techniques, as these are not core or essential elements of a stress reduction approach, while it is important for the RCT to adhere to NICE-recommended standards, which indicate that 4 sessions are optimal when group sessions are delivered by experienced facilitators. The program will be delivered by 3 experienced facilitators (2 psychological well-being practitioners and 1 psychiatrist) across the SRP groups recruited across all sites, ensuring consistent delivery of the intervention across all participating NHS sites.

#### Intervention Compliance

Intervention adherence, compliance rates (ie, session attendance), and any other data specific to the intervention, where applicable, will be collected electronically via a password-protected Microsoft Online Form, as per routine therapy protocol during the MBCT-L and SRP interventions. Retention rates and turnover by randomized group will be determined after the first recruitment wave. We will consider participants as compliant if they have attended 50% of the sessions on either intervention. Participants will not be allowed to continue with the intervention if they miss the first couple of sessions in either intervention arm. Participant attendance will be recorded by the facilitators for each intervention, as per routine practice, and reported to the project administrator, who will monitor attendance and contact participants if they miss the first or second session. Home practice adherence will not be monitored or recorded; as per routine practice, participants will be strongly encouraged to engage in home practice outside the formal weekly sessions; however, a potential lack of daily commitment to practice should not affect participants’ session attendance. Practitioners or facilitators will prompt participants to complete their daily home practice between intervention sessions and remind them of the upcoming intervention sessions.

### Outcome Assessments

#### Overview

Participants who provide consent will be required to complete the outcome assessments via web-based surveys through REDCap, which will contain the primary and secondary measures at baseline (before the intervention); up to 2 weeks before randomization; and at follow-up time points at weeks 6, 12, and 20 after randomization. Participants in both arms will receive the same outcome measures. Participants will be able to save their responses and return to the survey to complete their assessments at a later point. For both incomplete baseline and follow-up surveys, participants will receive 3 automated email reminders through REDCap asking them to complete the survey. If the survey is still incomplete, the research team may then send out 1 further personalized email reminder from the study email address.

#### Primary Outcome

The primary outcome will be the change in the PSS-14 [24] score from baseline to 20 weeks after randomization. The PSS-14 was selected as the primary outcome measure as it is a well-validated and internationally used self-report scale of perceived stress and has been widely used in previous trials assessing changes in stress levels in diverse populations, including public sector workers.

#### Secondary Outcomes

All secondary outcome measures used in the trial are widely recognized as valid and effective scales in assessing well-being, mental health state, and work-related engagement and performance in various populations, including health care workers. Although these measures are relatively brief to administer, they present limitations due to their reliance on self-report, which can entail biases, such as recall bias, and may not index the full complexity of an individual’s experience of stress or poor well-being. Secondary outcomes will all be collected at 6, 12, and 20 weeks after randomization. These standardized measures have been commonly used in intervention studies to assess changes in well-being, mental health state, work-related satisfaction, engagement, and performance (Textbox 2 [24,28-33]).

**Secondary measures used in the trial.**

- Perceived Stress Scale-14 [24]: This is a self-report scale that assesses the degree to which the respondent has perceived situations in their life as stressful within the past month. Responses are obtained through 14 items requiring ratings of statements from 0 (never) to 4 (very often). The assessment at 6 and 12 weeks will be treated as secondary outcomes.
- Generalized Anxiety Disorder-7 [28]: This is a 7-item instrument used to measure or assess the severity of generalized anxiety disorder. Each item asks the individual to rate the severity of their symptoms over the past 2 weeks. Response options range from 0 (not at all) to 3 (nearly every day).
- Patient Health Questionnaire-9 [29]: This is a 9-item scale used to measure the severity of depression. Each item asks the individual to rate how often they have been bothered by the listed symptoms over the past 2 weeks, with responses ranging from 0 (not at all) to 3 (nearly every day).
- The International Trauma Questionnaire [30]: This is a brief measure of posttraumatic stress disorder and complex posttraumatic stress disorder. Respondents are asked to think of an experience that troubles them the most and indicate how much they have been bothered by that experience in the past month by responding to 18 questions on a scale from 0 (not at all) to 4 (extremely). Questions refer to ways people typically feel, think about themselves, and relate to others.
- Five Facet Mindfulness Questionnaire [31]: The questionnaire items assess 5 aspects of mindfulness and their impact on well-being. Participants are required to rate statements on a 5-point scale from 1 (never or very rarely true) to 5 (very often or always true) on facets of mindfulness relating to describing, observing, nonreacting, acting with awareness, and nonjudging.
- Copenhagen Burnout Inventory [32]: This measures personal and occupational burnout in 19 items, including overall physical and psychological fatigue (6 items), physical and psychological fatigue related to work (7 items), and client-related burnout (6 items). Answers include “always, often, sometimes, rarely, and never or almost never” or “to a very high degree, to a high degree, somewhat, to a low degree, and to a very low degree.” The possible score range for the burnout scales is 0 to 100 (response options are coded in scores of 100, 75, 50, 25, and 0). Higher scores indicate a higher degree of exhaustion.
- Utrecht Work Engagement Scale-9 Item [33]: This measures 3 dimensions of work engagement, including vigor (3 items), dedication (3 items), and absorption (3 items). Items are presented as statements to which persons respond on a 7-point scale, with anchors 0 (never) and 6 (always or every day).

#### Economic Evaluation

We also plan to conduct an economic evaluation from the perspective of the NHS and personal social services.

#### Measures of Cost-Effectiveness

The primary measure of cost-effectiveness includes the incremental cost-effectiveness ratio (ICER) per quality-adjusted life year (QALY) gained for the MBCT-L versus SRP interventions.

Secondary measures include ICER per reduction in PSS-14 scores for MBCT-L versus SRP.

The following outcome data will be collected:

- Health-related quality of life outcomes will be collected at baseline and 6, 12, and 20 weeks after randomization. These will be measured via the widely used EQ-5D-5L [34].
- A health economics assessment adapted from the Client Service Receipt Inventory [35], which has been successfully used in a wide variety of studies of mental health, community-based health, and social care services, will be conducted. A mental health customized version will be administered to collect self-reported resource use for 6 months preceding data collection at baseline, 6 weeks preceding data collection at 6-week follow-up, 6 weeks preceding data collection at 12-week follow-up, and 8 weeks preceding data collection at 20-week follow-up. It is intended that the data gathered from these measures will be used for a cost-effectiveness analysis, subject to trial outcomes, should MBCT be superior to SRP.

#### Other Data Acquisition

We will aim to collect demographic data, including information about protected characteristics (eg, age, gender, ethnicity, pregnancy, marital status, highest qualification, religion, sexual orientation, disability, first language, caring responsibilities, and refugee and asylum seeker status). We will also gather job-related information on the job sector, job title, duration in the post, monthly salary, presenteeism, absenteeism, turnover intention, and service use (including well-being programs, primary care, outpatient appointments, inpatient appointments, private therapy, and medication), and, where available, practitioner or facilitator feedback data. Furthermore, data will be collected on recruitment uptake or rate; session attendance; compliance with treatment; any adverse events (AEs); attrition or dropout rates (in between intervention and loss to follow-up); and reasons for attrition or noncompliance, including diversion and inclusion barriers to intervention uptake and implementation.

#### Qualitative Study

A subsample of 30 participants (n=15, 50% in each intervention arm) will be recruited for the qualitative study after 20-week randomization (within an 8-week data collection window). The semistructured interviews will inquire into participants’ perceived changes after intervention and their experience and views in relation to intervention uptake and acceptability. They will be conducted online via Microsoft Teams and will be video or audio recorded for transcription purposes, depending on the participant’s preference for keeping the camera on or using the audio only.

#### Qualitative Study Outcomes

Semistructured interview data will elicit the following qualitative outcomes from participants:

- Perceived changes in their well-being and quality of life (ie, beneficial impacts and adverse effects)
- Perceived changes in their levels of work satisfaction, engagement, and performance (ie, beneficial impacts and adverse effects)
- Nature of well-being support sought and whether the given intervention has met their expectations
- Barriers and drivers to intervention route to uptake, attendance, engagement, and adherence (including aspects related to the intervention delivery and content or structure, personal or work circumstances, and managerial support)
- Recommendations for future improvement of intervention design and implementation

### Dissemination

We will report the trial findings and recommendations on the digital intervention product refinements to our PPIE group and other stakeholders, scientific audiences, the NHS confederation, NICE, networks of nursing and medical directors of NHS organizations, social care directors, Academic Health Science Network-East Midlands, NHS England (previously known as Health Education England), and professional well-being practitioner groups who will be required to staff and deliver these interventions.

### AEs in the Study

The structured context of MBCT-L and SRP includes psychoeducational materials or resources on how to manage distressing experiences arising during meditation or at other times in practice. The trained practitioners or facilitators delivering these interventions have a mechanism in place as routine practice for providing tailored advice and support at any stage. Once participants have commenced the intervention, they are prompted to report any AEs, emotional or physical, to the practitioner or facilitator. This will trigger a consultation meeting between the participant and the therapist team, who will provide advice and refer the participant to further support as appropriate (within the mental health well-being team, general practice, or mental health services). Participants are prompted to make adjustments during the meditation exercises to reduce any bodily discomfort and exert physical effort at a level that is comfortable for them during any body-moving exercises. Participants are also signposted in the PIS to available well-being support resources that they can access outside the trial.

AEs may occur and may or may not be linked to the intervention. These can include exacerbated emotional reactions, an increase in frequency or intensity of a preexisting (mental or physical) episodic event or illness, and worsening of symptoms present at baseline following the start of the study. Any AEs will be assessed for seriousness, expectedness, and causality. The causal relationship of an AE to the study intervention is assessed by the chief investigator in consultation with medical experts of the research team as “possible,” “probable,” or “definite” and will be reported as an AE in the study log and the ARC-EM Scientific Committee. Participants will be asked to contact the practitioner leading their intervention group or the study site immediately in the event of an AE. Any AEs will be recorded and closely monitored until resolution, stabilization, or it has been shown that the study treatment or intervention is not the cause. The chief investigator shall be informed immediately of any AEs and shall determine the seriousness and causality in conjunction with the therapists and the medical practitioners who are part of the research team. These will be recorded in the study log and instigate further investigation and follow-up if or as appropriate. The chief investigator shall be responsible for all AEs reported to the ARC-EM Scientific Committee and Ethics Committee Board.

### Data Management Plan

Source data will include participant demographic data, consent, assessment data logged by participants and the research or study team on the REDCap system, as well as any other supplementary data logged by the research or study team. Source data will also include participant interview transcripts. Access to the research data will be limited to assigned trial staff and researchers to allow trial-related monitoring and audits and ensure compliance with data management regulations. All data will be stored on secure University of Nottingham platforms, that is, in access-restricted, password-protected online research folders on the University of Nottingham OneDrive (Microsoft Corporation). Access will be restricted by user identifiers and passwords (encrypted using a 1-way encryption method). Research data will be anonymized by allocating a unique trial-specific number or code in the database. It will be ensured that identifiable data will be stored separately from research data and that the researchers do not have access to the identifiable or randomization data to retain blinding.

### Interview Data and the Use of Videoconferencing

Only platforms approved by the University of Nottingham (ie, Microsoft Teams) will be used for conducting interviews, as these platforms are encrypted. To retain privacy and confidentiality, researchers will ensure that they are working in an area where conversations cannot be overheard and the computer screen cannot be observed. Researchers will not use personal accounts or devices to contact participants. Interviews will be recorded on Microsoft Teams to facilitate transcription; interview recordings will be deleted from Microsoft Teams as soon as they are transcribed. The interview recordings will be pseudonymized, and any identifying information will be redacted in transcripts. Interview recordings and transcripts will be saved on access-restricted, password-protected online research folders on the University of Nottingham OneDrive.

The trial will follow the electronic data management policy and procedures of the University of Nottingham (sponsor) in accordance with the Data Protection Act 2018.

### Analyses

#### Statistical Method

All analyses will be conducted on an ITT basis; a description of the planned ITT and sensitivity analyses for both primary and secondary outcomes conforming to the estimand framework [36] has been provided in Multimedia Appendix 2. After exploratory analysis, multilevel modeling will be implemented to quantify the treatment effect estimate and its precision over the follow-up time points, with the treatment implementation group and each participant as a higher-level analytic unit and baseline measure, allocation status, follow-up time point, and interaction of follow-up×allocation as covariates with fixed effects. A similar analytic approach will be used to quantify the treatment effects on all secondary outcomes. Missing values will be explored and imputed using a multiple imputation approach under the missing at random assumption for the missingness mechanism [37]. Sensitivity analyses will be performed to explore the robustness of the treatment effect estimate, sensitive to the influence of data missingness, various limitations of the data, assumptions, and analytic approaches to data analysis. The latest available version of Stata (StataCorp) will be used for data analysis. A detailed trial statistical analysis plan setting out the proposed analyses has been published [38].

#### Economic Analysis

The initial estimates of cost-effectiveness will require a base case analysis before exploring the impact of uncertainty. To perform the base case analysis, first, each individual participant within the trial will have an estimated intervention cost, a health care cost, a QALY score based upon their EQ-5D-5L responses, and a PSS-14 score. Second, using ordinary least squares regression, incremental costs, QALYs, and PSS-14 scores will be estimated for MBCT-L versus SRP groups. These incremental values will then be used to estimate the ICER per QALY gained and the ICER per PSS-14 reduced. The base case analysis will use data from all participants who report complete data; however, a secondary analysis using all participants’ data will be conducted, with missing data controlled for using multiple imputation [37].

The base case analysis will not include any productivity costs (ie, due to absence from work), as per NICE guidelines [39]. A secondary analysis will be conducted, whereby productivity costs will also be included, using a similar approach as the base case analysis. All analyses will be conducted using the latest version of Stata. Multimedia Appendix 3 provides a detailed economic analysis plan.

#### Qualitative Data Analysis

On the basis of the expected nature of the data as per previous studies, thematic analysis is intended to be applied to the transcribed data from the semistructured interviews. The analytic approach is anticipated to be reflexive, guided by key principles of the grounded theory framework and the guidelines by Braun and Clarke [40,41] following their 6-step thematic analysis methodological approach. The latest version of NVivo (Lumivero) software will be used for coding. The process of codebook development will be adopted until a code agreement has been reached, and a reflexive log will be kept to document coders’ personal reflections and observations emerging during the coding and thematic analysis process [41], which will be used as a tool for minimizing potential bias in interpretation. A codebook example will be provided following the guidelines of Boyatzis [42], illustrating how codes were generated from participant quotes and how codes, in turn, led to the derivation of themes and subthemes. Interrater reliability between 2 raters will likely be determined based on the formula suggested by Miles and Huberman [43], which is suitable for small-scale participant samples and is defined as the number of agreements or the number of agreements and disagreements, with 75% being the minimum percentage to indicate adequate levels of agreement [44].

## Results

Recruitment of participants commenced on August 29, 2023. The target recruitment of 260 participants was reached on April 30, 2024. Follow-up outcome data collection was completed on September 30, 2024. We anticipate data analysis to be complete by July 2025.

## Discussion

### Anticipated Findings

There has been increasing recognition of the importance of well-being in the public sector workplace and its association with individual and work-related outcomes [5-7]. In light of the disproportionately high rates of psychological distress in health care, public care, and other employees in the public sector [1], well-being support provision offered within organizational settings is conducive to maintaining a healthy and effectively productive workforce. The overall aim of this trial is to determine whether an online mindfulness-based variant, MBCT-L, is superior to an online stress reduction approach, SRP, in reducing perceived stress and improving other mental health and job-related outcomes in health care and other public sector employees. Both interventions are recommended by NICE as effective psychotherapies for improving psychological well-being in public sector employees at risk of poor mental health in the context of individual-level therapies that can reduce stress levels and improve general psychological well-being. While the two approaches differ in their nature, structure, and program duration, this study is the first to directly compare these two types of interventions for their effectiveness and cost-effectiveness, being delivered in online modalities. Beyond the primary outcome of perceived stress, the trial will secondarily assess intervention change on other aspects of mental well-being (eg, anxiety, depression, posttraumatic stress, and burnout), which have been found to be affected in these cohorts, as well as work-related outcomes, such as work engagement, satisfaction, and performance, which are factors linked to presenteeism, absenteeism, and turnover in the workplace [5-7].

Furthermore, the trial will collect additional data through the web-based survey, which will include, among other data, information on work engagement and turnover intention, absenteeism, and previous uptake of well-being support within or outside the work settings, to allow us to describe the mental health state and needs of the given cohort. The NICE committee also stressed the potential resource impact of rolling out such interventions in the workplace sectors. Moreover, we will aim to conduct a budget impact analysis to inform on intervention cost in practice, although this will depend on the outcomes of the trial in terms of the intervention’s clinical effectiveness and cost-effectiveness.

While NICE recognizes that organizational-level and managerial-level policies and practices are important to address work-related issues, particularly in ensuring equality and work satisfaction, the committee has highlighted that local commissioners and health care providers have a responsibility toward enabling staff at risk of poor well-being to take up such well-being interventions in the workplace, within a supportive organizational culture and climate [8]. This is of particular importance, given the long-term impact of the COVID-19 pandemic on public care staff and considering its particular impact on individuals from Black, Asian, and minority ethnic communities and deprived socioeconomic backgrounds [45]. Preliminary reports from the NHS CHECK study, one of the largest ongoing cohort studies in the United Kingdom launched during the pandemic to longitudinally investigate the psychosocial impacts of COVID-19 on NHS staff, indicated that women, younger staff, and nurses faced poorer mental health outcomes than other health care staff and that NHS staff from ethnic minority communities faced significant workplace inequalities leading to negative health outcomes [46]. This trial will collect information on protected characteristics and recruitment uptake, session attendance and treatment compliance, attrition and dropout rates, and reasons for attrition and noncompliance. This includes barriers to intervention access, uptake, and engagement that may reflect issues related to equality, diversion, and inclusion, which will be explored in more depth via semistructured interviews in the qualitative substudy.

Considering that interventions that are perceived to tackle mental health can be heavily stigmatized in some cultures [47], a limitation of this study might be the potential lack of engagement by individuals of certain sociocultural backgrounds. We will emphasize in the trial that both interventions are intended to help people cope more effectively with stress in the workplace and busy personal lives in an attempt to address such stigma. Advertising will be wide and will be aimed at reaching all staff groups within the target cohort. We will keep engaging in discussions with PPIE representatives; equality, diversion, and inclusion representatives at NHS Trust; or well-being champions about any particular approaches within the set study design that may help in engaging staff from different ethnic groups. Our lead therapist team has also examined the content of the trial interventions to ensure that they are culturally sensitive to equality and diversity issues; however, the anticipated qualitative input by participants on their experience with and acceptability of the interventions, gathered via the semistructured interviews, will contribute to refining the interventions toward improved future practice. Along similar lines, the inclusion of selected NHS sites across the United Kingdom in this study represents a limitation of the study design and the representativeness of the data. Moreover, the measures used in the trial may pose a challenge to the interpretation of results, given their self-report nature and potential biases in recall or response bias.

A further shortcoming integral to the end point of assessing the superiority of MBCT-L is the lack of its wide availability across the NHS trusts in the United Kingdom compared to the readily available SRP programs. To overcome this limitation and use the digital intervention modality, we have adopted a study design that allows NHS trusts in the United Kingdom, where MBCT-L is not available, to partake in the trial as “channeling” sites, allowing the recruitment of eligible staff on either of the 2 interventions delivered through the 4 main NHS trust sites in the trial. It is hoped that this approach will also help mitigate potential low recruitment rates or loss to follow-up.

Possible unintended outcomes might include adverse effects following engagement with the interventions. Currently, relatively little has been reported on the potential adverse effects of MBCT in nonclinical populations. However, in a recent systematic review of AEs in meditation practices and meditation-based therapies, including relaxation and MBCT exercises [48], it was found that adverse effects during or after meditation practices occurred in nonclinical populations too and were associated with meditation practices, particularly anxiety and depression. The overall prevalence of meditation AEs was 8.3% in 55 studies, reporting at least 1 adverse effect, and this percentage is similar to those reported for psychotherapy practice in general. The structured context of MBCT and SRP includes psychoeducational materials and resources on how to manage distressing experiences arising during meditation or at other times, while there is a mechanism in place in the trial for the participant to consult the therapist in the event of excessive discomfort or distress or to be directed to other well-being support. This trial will log any adverse effects reported during the intervention period and at follow-up, with plans in place to review these events and the conduct of the trial with the National Institute of Health and Care Research ARC-EM Scientific Committee and the external steering committee as well as to terminate a participant’s engagement in the trial or the trial itself, should the criteria for doing so be fulfilled.

### Conclusions

The outcomes of this trial are expected to inform the NHS and other public care services as to which intervention might be more effective to be commissioned for provision to their staff who access such well-being interventions through the integrated care pathways of the NHS trust or other organizational well-being resource channels. Furthermore, we aim to inform NICE in light of its review findings that did not include any evidence of online MBCT or any studies comparing the effectiveness of MBCT versus SRP approaches. In terms of impact, we hope that there may be some immediate benefits not only to the staff receiving the interventions but also to those NHS services and other public care areas that already provide these interventions in the United Kingdom and internationally, so that they can refine their practices.

## Appendix

Multimedia Appendix 1 SPIRIT checklist.

Multimedia Appendix 2 Estimand framework.

Multimedia Appendix 3 Economic analysis plan.

## Abbreviations

AE: adverse event
ARC-EM: Applied Research Collaboration East Midlands
CONSORT: Consolidated Standards of Reporting Trials
ICER: incremental cost-effectiveness ratio
ITT: intention-to-treat
MBCT: Mindfulness-Based Cognitive Therapy
MBCT-L: Mindfulness-Based Cognitive Therapy for Life
NHS: National Health Service
NICE: National Institute for Health and Care Excellence
PIS: participant information sheet
PPIE: patient and public involvement and engagement
PSS-14: Perceived Stress Scale-14
QALY: quality-adjusted life year
RCT: randomized controlled trial
REDCap: Research Electronic Data Capture
SPIRIT: Standard Protocol Items: Recommendations for Interventional Trials
SRP: Stress Reduction Psychoeducation

## Data Availability

The deidentified quantitative datasets generated or analyzed during this study will be made available in the University of Nottingham Data Repository [49]. Deidentified minimal qualitative data, including quotes from across the participant sample and the corresponding codes, will also be shared on this repository in the form of a codebook. The generated participant interview transcripts will not be made publicly available due to potential breaches of participant confidentiality, as the transcripts are expected to contain personal and sensitive material.
