# Supplementary material for: Mindfulness-Based Cognitive Therapy for Life (MBCT-L) Versus Stress Reduction Psychoeducation (SRP) for the Improvement of Mental Well-Being in Health Care and Other Public Sector Staff: Protocol for the Well at Work Randomized Controlled Trial
Source: JMIR Res Protoc. 2025 May 26;14:e67695. doi: 10.2196/67695 (PMC12149769; doi:10.2196/67695)
Supplement: Multimedia Appendix 2 [file resprot_v14i1e67695_app2.docx]

# Multimedia Appendix 2: Description of planned statistical analyses for the MBCT outcome conform the ‘estimands’ framework

# Primary outcome: Change on PSS from baseline at 20 weeks post randomization (intention-to-treat)**^§^**

| **Objective:** To assess the superiority outcome of MBCT-L vs. SRP in change on perceived stress (PSS-14) from baseline at 20 weeks post randomisation. | |
| --- | --- |
| **Estimand:** The primary estimand is the mean difference in change from baseline to week 20 in PSS-14 between all participants assigned to MBCT-L or SRP including the effects of due to any intercurrent events | |
| **Treatment:**  A Mindfulness-Based Cognitive Therapy-for Life (MBCT-L) programme lasting 9 consecutive weeks, 8 two-hour weekly sessions with a gap week across the 9 weeks plus a top-up 9th session in week 10 which will be a retreat day (approx. 0.5 day).  A Stress-Reduction Psychoeducation (SRP) programme lasting 4 consecutive weeks, consisting of 4 two-hour weekly sessions.  Both interventions will be delivered online via Microsoft Teams and will require daily home practice (30-45 mins). Both interventions will start in the same week for the purposes of synchronising the follow-up data collection. | |
| ESTIMAND | ANALYSIS |
| **Target population** | **Analysis set** |
| Public sector staffs who are working in healthcare, social care and teaching. | All participants meeting eligibility criteria and being randomized. Participants assigned to MBCT-L programme through randomization will be the index treatment group; Participants assigned to SRP programme will be the comparator group. |
| **Variable** | **Outcome measure** |
| Change in perceived stress level 20 weeks after randomisation compared to baseline. | Change in perceived stress, measured with PSS-14 at baseline and 20 weeks post randomisation. |
| **Handling of intercurrent events** | **Handling of missing data** |
| - Allocated treatment not applied (treatment policy strategy, ie, include in analysis according to the treatment arm randomized). - Premature treatment discontinuation (treatment policy strategy) - Switch to other treatment arm before primary endpoint (treatment policy strategy). - Take extra non-study treatment before primary endpoint (treatment policy strategy). - Not attending enough treatment session (treatment policy strategy). - Further intercurrent events such as death are not anticipated in this study. if any in future, composite strategy will be used instead. | All missing primary endpoint will be imputed through analytical modelling by means of MCMC approach. |
| **Population-level summary measure** | **Analysis approach** |
| Mean difference in change in PSS-14 between the two arms at 20 weeks post randomisation. | Treatment effect estimate and its precision (95% confidence interval, 95%CI) on primary outcome outcomes measure will be quantified by multilevel modelling (MLM) with participants and treatment group as higher-level analytical units with covariate sets including baseline measure, binary group status, follow up time, interaction term of group and time.  Sensitivity analysis and secondary analysis for primary outcome were planned, see details in SAP section 5.2.3 & 5.2.4. |

**^§^** Sensitivity analysis and secondary analysis for primary estimand will be conducted by running same analytical modelling on per-protocol sample set, observed data only and safety dataset if the latter is different from observed dataset, and CACE analysis.

Table 2: Secondary outcome: Change on secondary outcomes from baseline at follow-up weeks post randomization (intention-to-treat)

| **Objective:** To assess the superiority outcome of MBCT-L vs. SRP in change on perceived stress (PSS-14) from baseline at 6 & 12 weeks post randomisation, change in GAD-7, PHQ-9,ITQ, FFMQ, CBI, UWES-9 from baseline at 6,12 and 20 weeks post randomisation. | |
| --- | --- |
| Estimand: The secondary estimands are the   1. Mean difference in change from baseline to week 6 & 12 in PSS-14 between all participants in two arms including the effects due to any intercurrent event; 2. Mean difference in change from baseline to week 6, 12 and 20 in GAD-7, PHQ-9, ITQ, FFMQ, CBI, UWES-9 between all participants in two arms including the effects due to any intercurrent event. | |
| **Treatment:**  A Mindfulness-Based Cognitive Therapy-for Life (MBCT-L) programme lasting 9 consecutive weeks, 8 two-hour weekly sessions with a gap week across the 9 weeks plus a top-up 9th session in week 10 which will be a retreat day (approx. 0.5 day).  A Stress-Reduction Psychoeducation (SRP) programme lasting 4 consecutive weeks, consisting of 4 two-hour weekly sessions.  Both interventions will be delivered online via Microsoft Teams and will require daily home practice (30-45 mins). Both interventions will start in the same week for the purposes of synchronising the follow-up data collection. | |
| ESTIMAND | ANALYSIS |
| **Target population** | **Analysis set** |
| Public sector staffs who are working in healthcare, social care and teaching. | All participants meeting eligibility criteria and being randomized. Participants assigned to MBCT-L programme through randomization will be the index treatment group; Participants assigned to SRP programme will be the comparator group. |
| **Variable** | **Outcome measure** |
| 1. Change in perceived stress level 6 and 12 weeks after randomisation compared to baseline. 2. Change in anxiety, depression, mindfulness, work engagement, burn out, and traumatic experience at 6, 12 and 20 weeks after randomisation compared to baseline. | 1. Change in perceived stress, measured with PSS-14 at baseline, 6 and 12 weeks post randomisation. 2. Change in anxiety (GAD-7), depression (PHQ-9), mindfulness (FFMQ), work engagement (uwes-9), burnout (CBIT), and traumatic experience (ITQ), measured at baseline, 6, 12 and 20 weeks post randomisation. |
| **Handling of intercurrent events** | **Handling of missing data** |
| - Allocated treatment not applied (treatment policy strategy, ie, include in analysis according to the treatment arm randomized). - Premature treatment discontinuation (treatment policy strategy) - Switch to other treatment arm before primary endpoint (treatment policy strategy). - Take extra non-study treatment before primary endpoint (treatment policy strategy). - Not attending enough treatment session (treatment policy strategy). - Further intercurrent events such as death are not anticipated in this study. if any in future, composite strategy will be used instead. | All missing secondary endpoint will be imputed through analytical modelling by means of MCMC approach. |
| **Population-level summary measure** | **Analysis approach** |
| 1. Mean difference in change in PSS-14 between the two arms at 6 &12 weeks post randomization. 2. Mean difference in change in GAD-7, PHQ-9, ITQ, FFMQ, CBI, UWES-9 between the two arms at 6, 12 & 20 weeks post randomization. | Treatment effect estimate and its precision (95% confidence interval, 95%CI) on each secondary outcome outcomes measure will be quantified by multilevel modelling (MLM) with participants and treatment group as higher-level analytical units with covariate sets including baseline measure, binary group status, follow up time, interaction term of group and time. |
