# Supplementary material for: Mindfulness-Based Cognitive Therapy for Life (MBCT-L) Versus Stress Reduction Psychoeducation (SRP) for the Improvement of Mental Well-Being in Health Care and Other Public Sector Staff: Protocol for the Well at Work Randomized Controlled Trial
Source: JMIR Res Protoc. 2025 May 26;14:e67695. doi: 10.2196/67695 (PMC12149769; doi:10.2196/67695)
Supplement: Multimedia Appendix 3 [file resprot_v14i1e67695_app3.docx]

**Assessing the cost-effectiveness of the Mindfulness-Based Cognitive Therapy for Life (MBCT-L) intervention versus Stress-Reduction Psychoeducation (SRP) for the improvement of mental wellbeing for healthcare, social care, and teaching professionals.**

**Background**

As public sector employees, healthcare staff usually experience disproportionately high stress levels especially the work-related stress, which was further exacerbated by COVID-19 pandemic. This will consequently result in negative individual-level outcomes, such as emotional labour, burnout, compassion fatigue and reduced quality of life; as well as negative organisational outcomes, such as poor job satisfaction, presenteeism, absenteeism and leaving work, including also poor care provision. Consequently, National Institute for Health and Care Excellence (NICE) recently recommended Mindfulness-based and stress-management training as effective individual-level psychotherapeutic interventions to be delivered in either online or face-to-face group modality to healthcare or other public care employees who have or are at risk of poor mental health. The NICE committee highlighted that local commissioners and healthcare providers have a responsibility towards enabling staff to take up some wellbeing interventions within a supportive organisational culture and climate. This is of particular importance given the ongoing impact of COVID-19, and especially in consideration of its impact on individuals from BAME or deprived socio-economic backgrounds.

While stress reduction psychoeducation (SRP) has been the standard usual care group programme offered widely to the National Healthcare Service (NHS) across all regions in England, Mindfulness-based Cognitive Therapy-for Life (MBCT-L) as a newer, third-wave intervention, based on the mindfulness approach and is becoming increasingly popular in NHS and other public sector services. Although the two approaches, SRP and MBCT-L, differ in their conceptual and practical premises, they have not yet been formally compared to one another in terms of their effectiveness as online programmes in improving stress and other wellbeing aspects and job-related outcomes. Therefore, a definitive randomised trial was needed to evidence the clinical effectiveness of MBCT-L vs SRP for the improvement of mental wellbeing in healthcare professionals as well as social care and teaching professionals. The two approaches differ in their duration, with MBCT-L typically being delivered as a 9-week programme (including a practice day) and SRP as a 4-week programme; while the delivery of MBCT-L requires more skilled staff. A health economics analysis is therefore required to assess the cost-effectiveness of MBCT-L versus SRP so as to inform organisations such as NICE and Health Education England, and public services and commissioning agencies that provide these interventions for their staff, on optimal future wellbeing service provision for public sector workers.

**Research Question**

Is Mindfulness-Based Cognitive Therapy for Life versus Stress-Reduction Psychoeducation cost-effective for the improvement of mental wellbeing for healthcare, social care, and teaching professionals?

**Aims**

*Primary:* Demonstrate the cost-effectiveness of Mindfulness-Based Cognitive Therapy-for Life (MBCT-L) verses Stress-Reduction Psychoeducation (SRP) for healthcare, social care, and teaching professionals.

*Secondary:*

- Estimate the average per participant cost of both the MBCT-L and SRP interventions
- Score EQ-5D-5l responses for both MBCT-L and SRP participants to estimate Quality-Adjusted Life Years (QALYs)
- Estimate incremental total costs and QALYs for MBCT-L verses SRP interventions
- Estimate the Incremental Cost-Effectiveness Ratio (ICER) per QALY gained for the MBCT-L verses SRP intervention
- Estimate the ICER per reduction in perceived stress for MBCT-L verses SRP using changes in the Perceived Stress Scale-14 (PSS-14)
- Determine the impact of uncertainty on the initial (basecase) evaluation findings

**Perspective**

NHS and Personal Social Services.

**Setting**

Four NHS England Trusts including Nottingham University Hospitals NHS Trust, Nottinghamshire Healthcare NHS Foundation Trust, Sussex Partnership NHS Foundation Trust, and Tees, Esk and Wear Valleys NHS Foundation Trust.

**Time horizon**

Short term within-trial. Follow-up length is 20 weeks. Due to the short time horizon, discounting is not to be done as all costs and outcomes are accrued within less than one year.

**Population**

Any individuals aged 18 years or older who are seeking access to well-being support and are a) part/full time or honorary/voluntary employment at one of the Trusts, b) currently in work (i.e. not on sickness absence, etc) c) competent command of verbal and written English language, and d) access to a stable internet connection. Exclusion criteria are a) concurrently attending or planning to attend a psychological or well-being programme in the next three months, b) current diagnosis of a mental health condition from a GP or specialist mental health professional, and c) experience of significant life events currently causing significant distress.

The trial aimed to recruit 260 participants across all four Trusts, with 130 participants in each arm of the trial.

**Comparator**

A Stress-Reduction Psychoeducation (SRP) programme which involves four two-hour weekly sessions, run over four consecutive weeks (five if there is a school break), delivered online via Microsoft Teams or Zoom. The SRP programme also requires 30-45 minutes of daily home practice.

**Experimental intervention**

Mindfulness-Based Cognitive Therapy-for Life (MBCT-L) programme which involves eight two-hour weekly sessions plus one half-day session of practice, all delivered online via Microsoft Teams or Zoom. The MBCT-L programme runs over nine consecutive weeks (10 if there is a school break), and requires 30-45 minutes of daily home practice.

**Utilities**

EQ-5D-5L questionnaires [1] are collected at baseline, 6, 12, and 20 weeks post randomisation. EQ-5D-5L scores are estimated using EQ-5D-5L value sets. [2] Quality-Adjusted Life Years (QALYs) will be estimated using the Area under the Curve (AUC) approach [3].

**Intervention / comparator costs**

For both MBCT-L and SRP, interventions costs will be estimated on a per participant level in 2022-2023 prices. For the interventions, costs are primarily associated with the facilitator. This includes the hourly salary of the facilitator, time spent delivering the sessions, time spent preparing for each session, time spent training facilitators, and the cost of providing a trainer. There is also the costs associated with the license for Microsoft Teams / Zoom software, which will be estimated on a cost per session.

As staff engage in the sessions of either programme at their convenience outside of work duties, there will be no cost associated with participant attending the session.

**Healthcare costs**

Healthcare costs will be estimated on a per participant level, estimated in 2022-2023 prices. Any primary care attendances (both with General Practitioner and Nurse Practitioner) will be costed using the Unit Costs of Health and Social Care Report. [4] Any secondary care costs will be estimated using NHS Reference Costs. [5] Any prescriptions offered will be estimated using the British National Formulary. [6] Any adverse events will also be costed using the above data sources.

A secondary analysis will include costs associated with productivity (i.e. absence from work). Productivity will be costed based upon participants reported band, taking the associated mid-point salary from NHS Pay Scales [7].

**Measures of Cost-Effectiveness**

Primary measure of cost-effectiveness: Incremental Cost-Effectiveness Ratio (ICER) per QALY gained for the MBCT-L vs SRP interventions.

Secondary measures: ICER per reduction in Perceived Stress Scale-14 (PSS-14 for MBCT-L versus SRP.

**Analytical approach**

The initial estimates of cost-effectiveness will require a basecase analysis before exploring the impact of uncertainty (see section below). To perform the basecase analysis, firstly each individual participant within the trial will have an estimated intervention cost, a healthcare cost, a QALY score based upon their EQ-5D-5L responses, and a PSS-14 score. Secondly, using Ordinary Least Squares Regression [3], incremental costs, QALYs, and PSS-14 scores will be estimated for MBCT-L verses SRP. These incremental values will then be used to estimate the ICER per QALY gained and ICER per PSS-14 reduced. The basecase analysis will use data from all participants who report complete data, however, a secondary analysis using all participants will be conducted, with missing data controlled for using multiple imputation [3].

The basecase analysis will not include any productivity costs (i.e. due to absence off work), as per National Institute of Health and Care Excellence Guidelines. [8] A secondary analysis will be conducted whereby productivity costs will also be included, using a similar approach as the basecase analysis.

All analyses will conducted using Stata 18 [9].

**Uncertainty**

Uncertainty will be explored using both deterministic one-way sensitivity analyses and probabilistic sensitivity analysis. One-way sensitivity analyses will be performed on:

- Incremental cost for MBCT-L verses SRP
- Incremental effectiveness for MBCT-L verses SRP in terms of both QALYs and PSS-14

Values will be varied between 0.5 and 2 times their basecase value. Results will be plotted on a Tornado Plot.

The probabilistic sensitivity analysis will use Non-Parametric Bootstrapping [10] to estimate pairwise incremental costs and outcomes (QALYs / PSS-14) and their associated ICERs. [3] 10,000 replications will be required. Based upon the probabilistic sensitivity analysis results, average values for incremental costs, outcomes, and ICERs will be estimated along with associated 95% confidence intervals. Results will be plotted on scatterplots of the cost-effectiveness plane, and cost-effectiveness acceptability curves. Finally, a value of information analysis will be performed to determine the expected value of perfect information [11].

**References**

1. EUROQOL. *EQ-5D-5L*. 2025 27/03/2025]; Available from: h<ttps://euroqol.org/information-and-support/euroqol-instruments/eq-5d-5l/.>

2. Dolan, P., *Modeling valuations for EuroQol health states.* Med Care, 1997. **35**(11): p. 1095-108.

3. Glick, H.A., et al., *Economic Evaluation in Clinical Trials*. 2nd ed. Handbook in Health Economic Evaluation. 2014: Oxford University Press.

4. Personal Social Services Research Unit. *Unit Costs of Health and Social Care programme*. 2025 27/03/2025]; Available from: h<ttps://www.pssru.ac.uk/unitcostsreport/.>

5. NHS England. *National Cost Collection for the NHS*. 2025 27/03/2025]; Available from: h<ttps://www.england.nhs.uk/costing-in-the-nhs/national-cost-collection/.>

6. National Institute for Health and Care Excellence. *British National Formulary (BNF)*. 2025 27/03/2025]; Available from: h<ttps://bnf.nice.org.uk/.>

7. NHS Employers. *NHS terms and conditions pay poster 2022/23 [Archived]*. 2022 27/03/2025]; Available from: h<ttps://www.nhsemployers.org/publications/nhs-terms-and-conditions-pay-poster-202223-archived.>

8. National Institute for Health and Care Excellence. *NICE process and methods: PMG9: Guide to the methods of technology appraisal 2013*. 2013 27/03/2025]; Available from: <https://www.nice.org.uk/process/pmg9/chapter/the-reference-case>.

9. StataCorp, *Stata Statistical Software: Release 18*. 2023, StataCorp LLC.: College Station, TX.

10. Briggs, A.H., D.E. Wonderling, and C.Z. Mooney, *Pulling cost-effectiveness analysis up by its bootstraps: a non-parametric approach to confidence interval estimation.* Health Econ, 1997. **6**(4): p. 327-40.

11. Wilson, E.C.F., *A Practical Guide to Value of Information Analysis.* PharmacoEconomics, 2015. **33**(2): p. 105-121.
